# Analysis of Colored Lesions of Chilli Yellow Ringspot Orthotospovirus Infection in Tomato Fruits

**DOI:** 10.3390/v17111426

**Published:** 2025-10-27

**Authors:** Yu Li, Jie Zhang, Kuo Wu, Yongdui Chen, Tiantian Wang, Zhongkai Zhang

**Affiliations:** 1 Biotechnology and Genetic Germplasm Resources Research Institute, Yunnan Academy of Agricultural Sciences, Kunming 650205, China; zhengjun2314@126.com (J.Z.); wk@yaas.org.cn (K.W.); yongduichen@126.com (Y.C.); wtt596061917@126.com (T.W.); 2 Key Laboratory of Agricultural Biotechnology of Yunnan Province, Kunming 650205, China

**Keywords:** CYRSV, tomato, colored lesion, chromoplast, transcriptome, metabolome, carotenoid, flavonoid

## Abstract

Chilli yellow ringspot orthotospovirus (CYRSV) causes colored lesions in tomato fruits. In this study, tomato fruits with different-colored lesions were used as experimental materials to explore the color formation mechanism. After CYSRV infection, the structure of chromoplasts inside the cells of tomato fruits was distorted and the number of plastoglobules per chromoplast significantly decreased, but the plastoglobule areas increased, as determined via ultrathin sectioning and transmission electron microscopy. Transcriptome and metabolome analyses revealed enrichment of secondary metabolites, carotenoids, and flavonoids in tomatoes with colored lesions. Metabolome analysis revealed markedly reduced carotenoid metabolites (lycopene and α-carotene) in both red-yellow and yellow tomatoes. The flavonoid metabolites rutin, catechin, and naringenin chalcone were markedly increased in the yellow group only. RT-qPCR analysis revealed that the expression of genes involved in carotenoid and flavonoid synthesis increased, but the expression of *C4H* decreased. Transcription regulators such as *AP2* and *MYB12* play important roles in flavonoid and carotenoid biosynthesis in CYRSV-infected tomato fruits. The results of greenhouse isolation experiments revealed that CYRSV may induce color lesions via decreases in plastoglobule numbers and chromoplast areas, the absence of carotenoid metabolites, and the presence of yellow-colored flavonoid metabolites. These results provide new insights into the interaction between CYRSV and tomato plants.

## 1. Introduction

Plant viruses can cause dwarfing, malformation, and fruit color changes (yellowing, chlorosis, or brown spots) in host plants by interfering with hormone or pigment synthesis. Viral infection may affect the synthesis of plant hormones (auxin, gibberellin, abscisic acid, etc.), resulting in accelerated or delayed flowering, chlorosis, dwarfing, and wrinkling [1,2]. However, the mechanism through which plant viral infection interferes with changes in fruit color remains unclear. Chilli yellow ringspot orthotospovirus (CYRSV) is a member of the genus *Orthotospovirus* in the *Tospoviridae* family of the order Bunyavirales and belongs to the evolutionary clade of watermelon silver mottle orthotospovirus (WSMoV) [3,4], which can cause yellow ringspot in pepper. We previously reported that CYRSV causes colored lesions on fruits after it infects tomato plants, but the underlying mechanism is unclear.

Colored lesions lower the quality of tomato, pepper, and melon products, resulting in severe economic losses. Tomato fruit color is primarily related to the accumulation and relative proportions of chlorophyll, carotenoid, and flavonoid pigments in the peel and pulp, which not only have high ornamental value but are also important indicators of the nutritional and medicinal value of the fruit. Carotenoids are a class of natural tetraterpenoid pigments that are widely distributed in plants, algae, fungi, and bacteria [5,6] and give many fruits and vegetables bright colors, such as red, orange, and yellow.

In tomato fruits, carotenoids exist mainly in the form of lycopene, lutein, and β-carotene and play important roles in fruit color changes and in the production of various plant hormones during the growth and development of tomatoes. They also affect the appearance and quality of tomatoes, thus affecting their commercial value. Moreover, carotenoids play an indispensable role in the regulation of human growth and development. Studies have shown that salinity stress can increase the content of carotenoids in tomatoes, and the use of saline soil has considerable potential to produce tomatoes with high levels of secondary metabolites (such as carotenoids and anthocyanins) [7].

Carotenoids exist in different plastids, including proplastids, amyloplastids, xanthosomes, chloroplasts, and chromoplasts, with the last being the main organelles involved in carotenoid biosynthesis and storage [8]. The carotenoids stored in chromoplasts can result in plant fruits, flowers, and roots of different colors, such as orange, red, and yellow. Most species contain more than one type of chromoplast. Chromoplasts are characterized by abundant plastoglobules (PGs), which are found in many plants, such as mangoes, yellow papayas, tomatoes, and oranges. An increase in the size/number of chromoplast compartments is closely associated with an increase in carotenoid accumulation [9]. However, analyses of the effects of viral infection on host pigment metabolism are yet to be performed.

Flavonoids are water-soluble pigments that are stored in the vacuoles of plant cells. The genes/metabolites related to the flavonoid metabolism pathway not only affect changes in fruit color but also increase disease resistance in some plants. Flavonoids are among the main pigments of plants and include anthocyanins (red, orange, blue, and purple pigments), chalcones and nerones (yellow pigments), and flavanols and flavones (white and pale yellow pigments), resulting in plants of a wide variety of colors [10]. Flavonoids are secondary metabolites with antioxidant and antibacterial functions; they are widely present in plant tissues and play important roles in plant physiology, including via antioxidant, anti-inflammatory, anticancer, antibacterial, antifungal, and antiviral effects. The silencing of the structural gene *DFR*, which is involved in the flavonoid metabolism pathway, significantly decreased the content of the disease-resistant substance catechin in poplar, and *CHI*, a key gene involved in flavonoid synthesis, significantly inhibited the germination of spores and the growth of mycelia of Dalichocladium [11]. The CHI activity of plants increased after exposure to pathogenic microorganisms, indicating that the flavonoid metabolism pathway regulated by CHI is one of the defense responses in plants against pathogens [12].

Therefore, the color of tomato fruits is related to the types and contents of carotenoid and flavonoid compounds present in the cells. In response to biotic and abiotic stresses, plants can regulate the biosynthesis of carotenoids. Research has been conducted on the carotenoid metabolic pathway [13,14,15,16] and flavonoid metabolic pathway [17,18] and biotic stress; however, the relationships between these pathways and biotic stress, especially viral stress, remain unclear. In recent years, CYRSV has become widespread in tomatoes in Yuanmou, Yunnan Province, where it causes yellowing, yellow ringspot, and other colored lesions [19]. However, the mechanisms through which viruses induce fruit color lesions and the connection between viruses and colored lesions have not yet been reported. Yuanmou is an important tomato production area in Yunnan. Understanding the mechanism through which CYRSV induces fruit color changes in tomato fruits in Yuanmou is crucial in the prevention and control of viral diseases in tomatoes in this region.

In this study, we collected tomato fruits with different-colored lesions. CYRSV accumulation was analyzed via conventional PCR and absolute RT–qPCR, and the distribution of virus particles within cells and the ultrastructural lesions of chromoplasts were observed via transmission electron microscopy (TEM). Differentially expressed genes and metabolites involved in the carotenoid and flavonoid metabolic pathways were analyzed via comparative transcriptomic and metabolomic analyses, respectively. The key genes and transcription factors associated with the carotenoid and flavonoid metabolic pathways were identified using RT–qPCR. We also performed correlation analysis to explore the relationships among CYRSV, key genes, and transcription factors. A CYRSV friction inoculation experiment was conducted on tomatoes in a greenhouse to observe the effect of CYRSV inoculation on the color lesions of tomato fruits. The results of this study provide essential data on the regulatory effects of CYRSV on the metabolic pathways of carotenoids and flavonoids in tomato fruits and provide a new method for the prevention and control of viral diseases.

## 2. Materials and Methods

### 2.1. Plant Material

Normal and CYRSV-infected ‘Liangsi’ tomato samples were sourced from three geographic locations in Yuanmou County, Yunnan Province, China (Xiaobingling, Yangjie, and Jinlong). The experimental materials were stored either at 4 °C or −80 °C until use. The tomato variety “Liangsi” was used in the infection experiments in this study. These plants were cultivated in an artificial climate chamber with the temperature maintained at 25 °C for 16 h of light and at 20 °C for 8 h of darkness each day.

### 2.2. Virus Strains and Inoculation

The CYRSV isolate utilized in this study was preserved at the Biotechnology and Germplasm Resources Institute, Yunnan Academy of Agricultural Sciences. The seedlings were inoculated with CYRSV when four leaves were fully expanded, and the appearance of a new leaf was noted. The CYRSV inoculum was prepared from infected leaves of *Nicotiana benthamiana*. One gram of infected leaves was ground in 5 mL of 0.02 M phosphate buffer with a pH of 7.0. *Nicotiana benthamiana*, *Chenopodium amaranticolor*, and tomato plants were dusted with 600-mesh carborundum and then inoculated by rubbing the leaves with the prepared inoculum. The plants were subsequently maintained in a greenhouse at temperatures ranging from 25 to 30 °C. Symptoms of CYRSV infection were initially observed seven days post inoculation.

### 2.3. Virus Identification

To identify single infection with CYSRV in tomato samples, we used both negative staining/transmission electron microscopy observations of fruit samples and reverse transcription–polymerase chain reaction (RT–PCR) with specific primers for the molecular detection of tomato virus disease. The four specific primers used were as follows: CYRSV-F: 5′-TCACACTTCCAGAGAAGAACTTGGT-3′; CYRSV-R: 5′-ATGTCTAACGTTAAGCAACTT-3′; TSWV-F: 5′-GTCAGTGCCTCCAATCCTGT-3′; TSWV-R: 5′-TGTCTGGAAAACATCGACCA-3′; TOBRFV-F: 5′-ATGTCTTACACAATCGCAACTCCA-3′; TOBRFV-R: 5′-TCAAGATGCAGGTGCAGAGGACC-3′; TZSV-F: 5′-TTAAAAAGACAGATCATTGCTGCTC-3′; and TZSV-R: 5′-ATGTCTAACGTCCGGAGTTTAA-3′.

### 2.4. Transmission Electron Microscopy (TEM)

To observe virus particles, tomato tissues were cut into thin shreds on a slide with 2.5% glutaraldehyde and then incubated for 2 min. Next, a drop of the glutaraldehyde mixture was blotted onto a clean sealing film, and the TEM grids were placed on the surface of the droplets for 2 min. The grids were washed briefly at pH 7.2 with phosphate-buffered saline and negatively stained with lead citrate and 1% phosphotungstic acid for 1.5 min. Subsequently, the grids were observed using a FEI TECNAI Spirit G2 transmission electron microscope (ThermoFisher Scientific, Waltham, MA, USA) at a magnification of 30,000×. Virus particles were observed from photographs taken from at least three randomly chosen fields per grid.

### 2.5. Chromoplast Ultrastructural Analysis

The tomato fruits were cut into 1 × 3 mm rectangular pieces and then fixed in 2.5% glutaraldehyde at 4 °C overnight. The tomato pieces were subsequently washed three times with 0.1 M PBS for 5 min each and then incubated in 1% (*w*/*v*) osmium tetroxide for 4 h. The samples were subsequently washed three times with 0.1 M PBS for 5 min each, dehydrated with different concentrations of ethanol, permeabilized, and embedded in Epon812 resin (SPI, Stouffville, CA, USA), which was then polymerized at 60 °C. The embedded mass was sliced into semithin sections to observe the location of the structures, and ultrathin sections were then cut with an ultramicrotome (EM UC7, Leica, Wetzlar, Germany) and collected onto Formvar-coated Cu grids (Electron Microscopy China, Beijing, China). The sections were stained with 2% (*w*/*v*) uranyl acetate for 15 min, followed by lead citrate for an additional 7 min. The sections were then observed and imaged with a transmission electron microscope (TECNAI G2, ThermoFisher, MA, USA). Each group consisted of three biological replicates. The number and area of chromoplasts were determined from photographs taken from at least ten randomly chosen cells per sample. The number and area of plastoglobules were determined from photographs taken from at least five randomly chosen chromoplasts per sample. The chromoplast and plastoglobule diameters were measured via TEM Imaging & Analysis (TIA) software version 4.7 SP3 (ThermoFisher, MA, USA).

### 2.6. Virus Accumulation Tests

The absolute fluorescence real-time RT–qPCR method was used to determine the copy number of the CYRSV *N* gene. The 224 bp target fragment was amplified via RT–qPCR using cDNA as a template with the primers CYRSV-N-qF (5′-GCGGTACTGCAGATGTTGAA-3′) and CYRSV-N-QR (5′-GGTCCAATCTTCTGGTCCAA-3′). Next, the target fragments were extracted using the AxyPrep^TM^ DNA Gel Extraction Kit (Axygen, Tewksbury, MA, USA) and ligated into the pMD19-T vector (TaKaRa, Beijing, China) to obtain the plasmid standard. The plasmid with the correct sequence was used as the positive control plasmid standard. The concentration of the plasmid was determined using a NanoDrop 2000 (Thermo Fisher, Waltham, MA, USA), and the CYRSV copy number was subsequently calculated using the following formula: copy number (copies/μL) = plasmid concentration (g/μL) × 6.02 × 10^23^/plasmid molecular weight. The calculated copy number of the plasmid standard was 0.94 × 10^11^. The plasmid DNA was then subjected to 10-fold serial dilutions (0.94 × 10^9^, 0.94 × 10^8^, 0.94 × 10^7^, 0.94 × 10^6^, 0.94 × 10^5^ μL, 0.94 × 10^4^ μL, 0.94 × 10^3^ μL, and 0.94 × 10^2^ μL) to prepare standards for absolute quantification. The above primers were used to detect CYRSV accumulation via 2× Accurate Taq mix (ROX) and a real-time RT–qPCR instrument (ABI QuantStudio 6, ABI, Carlsbad, CA, USA). A total of 2 μg of total RNA from each sample was extracted and reverse-transcribed to cDNA, which was subsequently diluted 10-fold. The number of copies of CYRSV in the unknown sample was determined by substituting the Ct value of the unknown sample into the resulting standard curve. Three replicates were established for each sample.

### 2.7. Reverse Transcription–Real-Time Quantitative PCR (RT–qPCR)

Total RNA extraction and reverse transcription were performed using a plant total RNA extraction kit and reverse transcription kit (Hunan Accurate Bioengineering Co., Ltd., Changsha, China). A total of 2 μg of total RNA per sample was reverse-transcribed to cDNA and then diluted 10-fold. Genes related to the carotenoid metabolism pathway (*SlZDS*, *SlZISO*, *SlPSY1*, *SlZEP*, *SlPDS*, *SlCRTISO*, *SlLCYB*, and *SlLCYE*) and flavonoid metabolism pathway (*SlPAL*, *SlC4H*, *Sl4CL*, *SlCHS*, *SlCHI*, *SlF3H*, and *SlFLS*) and transcription factors regulating pigment metabolism (*SlAP2α*, *SlMYB12*, and *SlPIF1α*) were determined via real-time quantitative PCR (ABI QuantStudio 6). The specific primers used for the qPCR were as follows: *SlZDS*-F: 5′-TTGGAGCGTTCGAGGCAAT, *SlZDS*-R: 5′-AGAAATCTGCATCTGGCGTATAGA-3′; *SlZISO*-F: 5′-TATGAGGATTACCAGGCATCC, *SlZISO*-R: 5′-ACAGCGTGTGAGCTAAGCAC-3′; *SlPSY1*-F: 5′-TGGCCCAAACGCATCATATA, *SlPSY1*-R: 5′-CACCATCGAGCATGTCAAATG-3′; *SlZEP*-F: 5′-TTGGGTTCTAGGAGGCAATG, *SlZEP*-R: 5′-CCCGCAGGTAAAAGTAACCA-3′; *SlPDS*-F: 5′-GTGCATTTTGATCATCGCATTGAAC, *SlPDS*-R: 5′-GCAAAGTCTCTCAGGATTACC-3′; *SlCRTISO*-F: 5′-TTTTGGCGGAATCAACTACC, *SlCRTISO*-R: 5′-GAAAGCTTCACTCCCACAGC-3′; *SlLCYB*-F: 5′-TCGTTGGAATCGGTGGTACAG, *Sl*LCYB-R: 5′-AGCTAGTGTCCTTGCCACCAT-3′; *SlLCYE*-F: 5′-GGATCAGCAGTCTAAGCTTTCTG, *SlLCYE*-R: 5′-CGATTACCACTAAATCCAGTACG-3′; *SlAP2a*-F: 5′-AGTGGGGAACAAAATTGACG, *SlAP2a*-R: 5′-TGCTGCATGTGCTGTATCAA-3′; *SlMYB12*-F: 5′-GCCAGCTTGTGATAGTGCCAT, *SlMYB12*-R: 5′-AAGGCTTCCCTTGGCCTCTA-3′; *SlPIF1a*-F: 5′-TCCATATCAGCAGTTTTTTGGTCTC, *SlPIF1a*-R: 5′-GCTGCTGCCGGATTTACTATTAC-3′; *SlCH4*-F: 5′-TCACGTCCACGTAACGTTGTG-3′, *SlCH4*-R: TGATACGTCTCATTTTTCTCCAATG-3′; *Sl4CL*-F: 5′-AACCCCACTGCTAAGGCTATTTT-3′, *Sl4CL*-R: GACAATTACCCCCAAATGTCCTAA-3′; *SlF3H*-F: 5′-CTGTTCAGCCCGTTGAAGGT-3′, *SlF3H*-R: 5′-ACCACTGCTTGATGATCAGCAT-3′; *SlFLS*-F: 5′-GAGCATGAAGTTGGGCCAAT-3′, *SlFLS*-R: 5′-TGGTGGGTTGGCCTCATTAA-3′; *SlPAL*-F: 5′-CAGCCTAAGGAAGGACTTGCA-3′, *SlPAL*-R: 5′-GAAAATCGCTGACAAGACTTCAGA-3′; *SlCHI*-F: 5′-GTGCTTCTGGGAGTGCAAAGA-3′, *SlCHI*-R: 5′-CCCTTGTTCCACCTAAGTACCATT-3′; *SlCHS*-F: 5′-TGAGCACAAGACTGAGCTCAAAG-3′, *SlCHS*-R: 5′-GGTTCTCTTTCAAGATTTCTTCGG-3′; *Slβ-actin*-F: 5′-CCTCAGCACATTCCAGCAG-3′, *Slβ-actin*-R: 5′-CCACCAAACTTCTCCATCCC-3′.

### 2.8. Detection of Carotenoid Metabolites

Carotenoid metabolites were analyzed using a UPLC system (ExionLC^TM^ AD, AB Sciex, Baltimore, MA, USA) and tandem mass spectrometry (MS/MS; OTRAP^®^6500+, AB Sciex, MA, USA) [20,21,22]. The samples were freeze-dried, ground into powder (30 Hz, 1.5 min), and stored at −80 °C until use. Fifty milligrams of powder was weighed and extracted with 0.5 mL of a mixed solution of n-hexane/acetone/ethanol (1:1:1, *v*/*v*/*v*). The extract was vortexed for 20 min at room temperature. The supernatants were collected after undergoing centrifugation at 12,000 r/min for 5 min at 4 °C. The residue was re-extracted by repeating the above steps again under the same conditions. The mixture was then evaporated to dryness and reconstituted in 150 μL of dichloromethane. The solution was filtered through a 0.22 μm membrane filter for further LC–MS/MS analysis.

The sample extracts were analyzed using a UPLC–APCI–MS/MS system (UPLC, ExionLC™ AD, https://sciex.com.cn/ accessed on 11 December 2024; MS, 6500 Triple Quadrupole (AB Sciex, Baltimore, MA, USA), https://sciex.com.cn/ accessed on 10 January 2025). The analytical conditions were as follows: UPLC column, YMC C30 (3 μm, 100 mm × 2.0 mm i.d.); solvent system, methanol/acetonitrile (1:3, *v*/*v*) with 0.01% BHT and 0.1% formic acid (A) and methyl tert-butyl ether with 0.01% BHT (B); gradient program starting at 0% B (0–3 min), increasing to 70% B (3–5 min), then increasing to 95% B (5–9 min), and finally returning to 0% B (10–11 min); flow rate, 0.8 mL/min; temperature, 28 °C; and injection volume, 2 μL.

Linear ion trap (LIT) and triple quadrupole (QQQ) scans were acquired via a triple quadrupole–linear ion trap mass spectrometer (QTRAP), QTRAP^®^ 6500+ LC–MS/MS System (AB Sciex, Baltimore, MA, USA), equipped with an APCI heated nebulizer, operating in positive ion mode and controlled using Analyst 1.6.3 software (AB Sciex, MA, USA). The APCI source operation parameters were as follows: ion source, APCI+; source temperature, 350 °C; and curtain gas (CUR) pressure, 25.0 psi. Carotenoids were analyzed using scheduled multiple reaction monitoring (MRM). Data acquisition was performed using Analyst 1.6.3 software (Sciex). Multiquant 3.0.3 software (Sciex) was used to quantify all metabolites. The mass spectrometer parameters, including the declustering potential (DP) and collision energy (CE) for individual MRM transitions were determined through further DP and CE optimization. A specific set of MRM transitions was monitored for each period according to the metabolites eluted within this period.

### 2.9. Detection of Flavonoid Metabolites

The flavonoid compounds were analyzed using a UPLC system (ExionLC^TM^ AD, AB Sciex, MA, USA) and tandem mass spectrometry (MS/MS; OTRAP^®^6500+, AB Sciex, MA, USA) [23]. After the samples were vacuum-freeze-dried, they were ground into powder using a ball mill (30 Hz, 1.5 min). A total of 10 μL of 4000 nM internal standard working solution and 500 μL of 70% methanol solution were added to a 20 mg powder sample, which was ultrasonicated for 30 min and then centrifuged at 4 °C for 5 min (12,000 rpm), after which the supernatant was removed. The sample was filtered through a 0.22 μm filter membrane and stored in an injection bottle for subsequent detection and analysis. The conditions used were as follows: chromatographic column, Waters ACQUITY UPLC HSS T3 C18 column (1.8 μm, 100 mm × 2.1 mm i.d.; Waters, Milford, MA, USA); mobile phase, phase A, ultrapure water (0.05% formic acid); phase B, acetonitrile (0.05% formic acid); flow rate, 0.35 mL/min; column temperature, 40° C; injection volume, 2 μL; and elution gradient, 0 min A/B (90:10, *v*/*v*), 1 min A/B (80:20, *v*/*v*), 9 min (30:70, *v*/*v*), 12.5 min (5:95, *v*/*v*), 13.5 min (5:95, *v*/*v*), 13.6 min (90:10, *v*/*v*), and 15 min (90:10, *v*/*v*). The mass spectrometry conditions were as follows: electrospray ionization (ESI) ion source temperature, 550 °C; mass spectrometry voltage in positive ion mode, 5500 V; mass spectrometry voltage in negative ion mode, −4500 V; and curtain gas (CUR) pressure, 35 psi.

### 2.10. Transcriptome Analysis

Total RNA extraction and reverse transcription were performed using a plant total RNA extraction kit and reverse transcription kit (Hunan Accurate Bioengineering Co., Ltd., Changsha, China). RNA integrity and purity were tested via agarose gel electrophoresis and a NanoDrop2000 (Thermo Fisher Scientific, Waltham, MA, USA), respectively. cDNA was synthesized and sequenced on the Illumina HiSeq platform from Tsingke Biotechnology Corporation (Kunming, China). The mapped reads were compared with the original genome annotation information to identify the original unannotated transcription region and to discover new transcripts and new genes in the species to supplement and improve the original genome annotation information. Differentially expressed genes (DEGs) were identified on the basis of their fragments per kilobase per million reads (FPKM). An adjusted *p* value ≤ 0.05 and log2 (fold change) (log2FC) ≥ 1 were used as criteria to identify the DEGs associated with the different treatments. Assembled contigs were annotated via the Basic Local Alignment Search Tool (BLAST) online software on the NCBI website against seven public databases—Nr, Nt, SwissProt, KOG, Pfam, GO, and KEGG—with >90% identity and an E value < 0.00001.

### 2.11. CYRSV Greenhouse Isolation Inoculation Experiment

‘Liangsi’ tomato seedlings were inoculated with CYRSV when 7–8 leaves were fully expanded. Tomato plants were dusted with 600-mesh carborundum and then inoculated by rubbing the leaves with PBS in the mock group and with the prepared CYRSV inoculum in the CYRSV group. Twenty tomato plants were included in each group. The plants were subsequently maintained in a greenhouse at temperatures ranging from 25 to 30 °C. When the tomatoes were ripe, the color of the fruit lesions was observed and recorded, the ultrastructures of the chromoplasts in the tomato fruit cells of the two groups were determined via transmission electron microscopy (TEM), and the changes in the contents of carotenoids and flavonoid metabolites in the tomatoes were detected.

### 2.12. Statistical Analysis

Statistical significance was determined using Student’s *t* test or one-way ANOVA via GraphPad Prism version 5.0a (GraphPad Software, San Diego, CA, USA). Student’s *t* tests were used to analyze the significant differences between two groups, one-way ANOVA was used to determine the significant differences among three groups, and Pearson correlation analysis was used to analyze the correlation between two genes. The experiments were performed at least three times.

## 3. Results

### 3.1. Color Lesions in CYRSV-Infected Tomato Fruits

**Figure 1:**
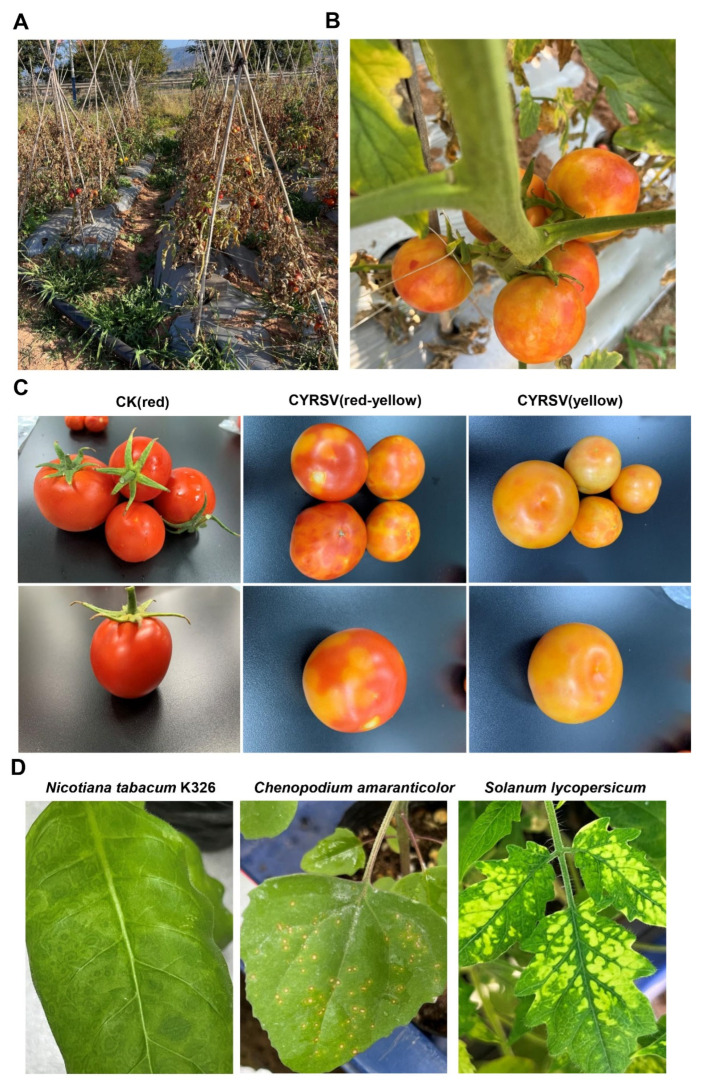
Color loss symptoms. (**A**) Field symptoms of CYRSV. (**B**) Tomatoes showing color changes. (**C**) Tomatoes showing lesions of different colors. (**D**) Yellow ringspot lesions appeared in *Nicotiana tabacum* K326, necrotic spot lesions appeared in *Chenopodium amaranti*, and wrinkled, necrotic spot lesions appeared in *Solanum lycopersicum* after inoculation with extracts from tomatoes with color lesions.

In recent years, CYRSV has caused serious damage to tomato cultivation in Yunnan Province (Figure 1A). Colored lesions on tomatoes were characterized by the presence of irregular yellow patches, yellow ringspot, and yellowing where a red pigment would normally have been present (Figure 1B). The tomatoes were classified into three groups: the CK (red) group (mature red tomatoes without symptoms), the CYRSV (red-yellow) group, and the CYRSV (yellow) group (Figure 1C). *Nicotiana tabacum* K326, *Chenopodium amaranticolor*, and *Solanum lycopersicum* were used to test for the presence of viruses in the colored lesions of tomato fruits collected from three different locations in Yuanmou County, Yunnan Province, China (Xiaobingling, Yangjie, and Jinlong). Tomato fruit extracts from all color-lesioned tomatoes caused the formation of yellow ringspot lesions in *N. tabacum* K326 leaves, withered spots in *Chenopodium amaranticolor*, and leaf curl and necrosis in tomato leaves at 9 d post inoculation (Figure 1D). In contrast, no lesions were observed when the tomato fruit extracts from control red tomatoes were used as the inoculum, indicating that the colored lesions were associated with the presence of a particular virus.

### 3.2. Colored Lesions Were Associated with the Presence of CYRSV in Tomato Fruits

**Figure 2:**
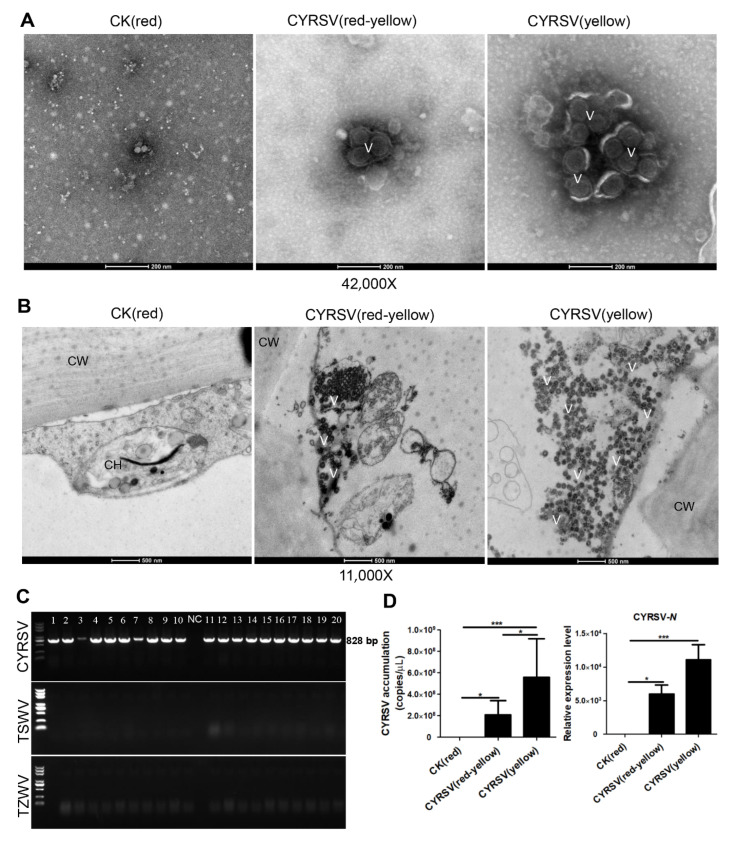
Identification of CYRSV in tomato fruits with colored lesions. (**A**) Viral particles present in fruit extracts from tomatoes with colored lesions were detected via negative staining and electron microscopy. Scale bar = 500 nm. (**B**) Ultrathin sections and electron micrographs showing viral particles present in the cytoplasm of pulp cells of tomatoes with colored lesions. V, virus; CW, cell wall; CH, chloroplast. Scale bar = 500 nm. (**C**) Results of electrophoretic analysis of tomato samples using specific primer PCR. Lane 1–20 indicate tomatoes with colored lesions. Healthy tomatoes were used as the NC. (**D**) CYRSV accumulation in tomato fruits in different groups was determined via absolute and relative RT–qPCR. The data are presented as the mean ± SD (*n* = 5), and one-way ANOVA was used to determine the significant differences among the three groups. Asterisk indicate significant differences: * *p* < 0.05, *** *p* < 0.001.

RT–qPCR and TEM were used to examine the quantity and morphological characteristics of the viral particles present in a subset of the colored lesion and asymptomatic control tomatoes. Negative staining and TEM revealed that the pulps of the tomato fruits that displayed colored lesions contained many spherical viral particles with diameters of 80–120 nm (Figure 2A). Ultrathin sectioning and TEM revealed that spherical viral particles with diameters of 80–120 nm were dispersed in the cytoplasm in the cells of the tomato fruits that displayed colored lesions (Figure 2B). To identify the type of spherical virus, we performed electrophoresis on the putative viral sequences amplified from the cDNA via RT–PCR with specific primers. The spherical viruses infecting the tomatoes were mainly of the genus *Orthotospovirus*, so specific primers for three important orthotospoviruses (TSWV, TZSV, and CYRSV) were used. The electrophoretic results were positive only for the CYRSV virus (Figure 2C); the PCR product was also confirmed to be CYRSV via sequencing. After absolute and relative quantification of CYRSV accumulation, the results revealed that CYRSV had accumulated in both the CYRSV (yellow) group and the CYRSV (red-yellow) group and that the CYRSV accumulation was greatest in the tomatoes of the CYRSV (yellow) group (Figure 2D). These results suggest that CYRSV infection can lead to different-colored lesions in fruits at the ripening stage and that these lesions are associated with the accumulation of CYRSV viral particles, with greater virus accumulation resulting in a lighter fruit color.

### 3.3. Ultrastructural Analysis of Chromoplasts in Tomato Fruits with or Without Color Lesions

**Figure 3:**
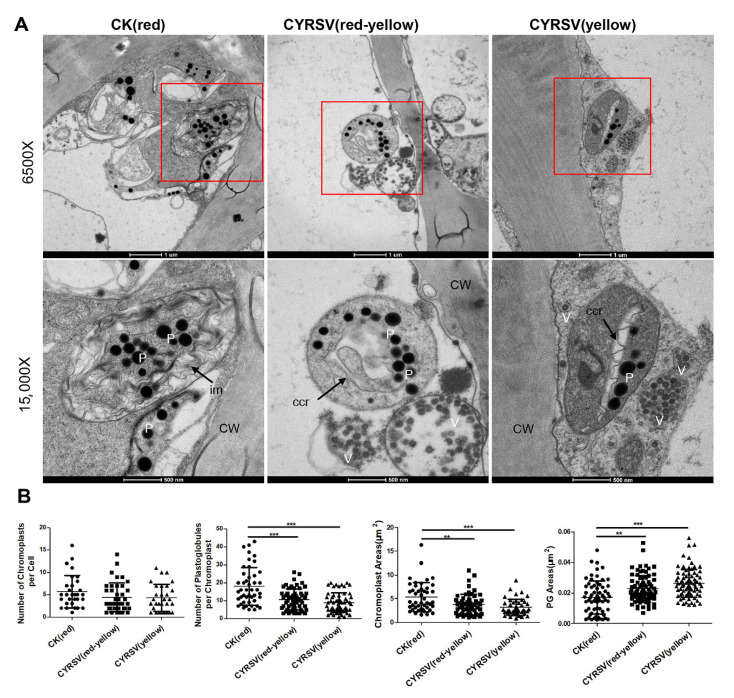
Transmission electron micrographs of chromoplasts present in tomato fruits with different-colored lesions. (**A**) Chromoplast micrographs of the CK (red), CYRSV (red-yellow), and CYRSV (yellow) groups. The lower image is a magnification of the red box in the upper image. P, plastoglobule; ccr, carotenoid crystalloid; CW, cell wall; V, virus; im, inner membrane. Scale bar = 1 μm or 500 nm. (**B**) Number and areas of chromoplasts and plastoglobules in tomato fruits in the CK (red), CYRSV (red-yellow), and CYRSV (yellow) groups. Asterisk indicate significant differences: ** *p* < 0.01, *** *p* < 0.001.

The ultrastructural characteristics of chromoplasts in tomato fruits with different-colored lesions were observed via ultrathin sectioning and TEM. As shown in Figure 3A, we clearly observed chromoplasts differentiated from chloroplasts, which contained black particles of varying sizes with high electron density, namely, plastoglobules. Viral particles were distributed around the chromoplasts in the CYRSV-infected tomato fruits (Figure 3A). The number of chromoplasts did not significantly differ among the CK (red), CYRSV (red-yellow), and CYRSV (yellow) groups, whereas both the chromoplast areas and the plastoglobule number significantly decreased after CYRSV infection; however, the plastoglobule areas significantly increased after CYRSV infection (Figure 3B). Furthermore, the area of the chromoplast and the number of plastoglobules were the lowest, and the area of the plastoglobules was the highest, in the CYRSV (yellow) group (Figure 3B). The results revealed that the chromoplast ultrastructure changed significantly in tomato fruits after CYRSV infection; thus, the altered structure of the chromoplast was associated with the presence of CYRSV in tomato fruits.

### 3.4. Metabolome Analysis

**Figure 4:**
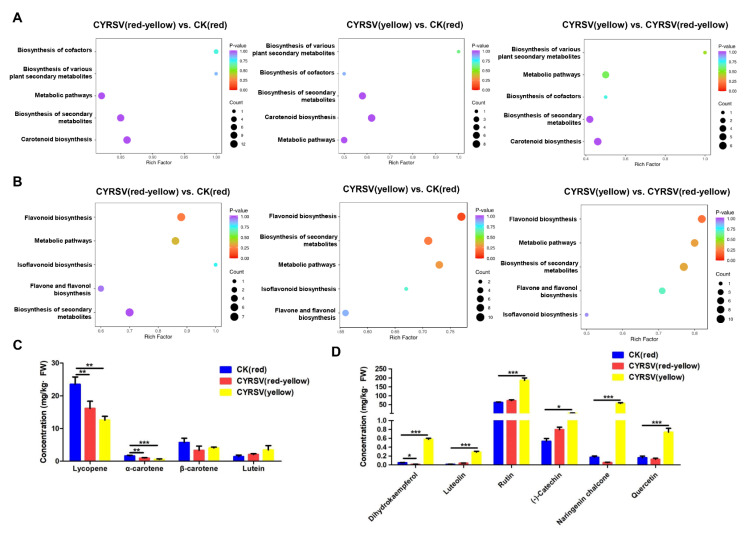
Analysis and concentrations of carotenoid and flavonoid metabolites in the fruits of different groups of tomatoes. (**A**) Kyoto Encyclopedia of Genes and Genomes (KEGG) enrichment map of carotenoid metabolites in three comparison groups: CYRSV (red-yellow) vs. CK (red), CYRSV (yellow) vs. CK (red), and CYRSV (yellow) vs. CYRSV (red-yellow). A higher enrichment factor indicates a greater degree of enrichment. (**B**) Differentially abundant metabolite enrichment pathways for flavonoids in three comparison groups: CYRSV (red-yellow) vs. CK (red), CYRSV (yellow) vs. CK (red), and CYRSV (yellow) vs. CYRSV (red-yellow). The color and size of the points represent the range of the *p* value and the number of differentially abundant metabolites enriched, respectively. A more intense red color indicates more significant enrichment. (**C**) Lycopene, α-carotene, β-carotene, and lutein contents of two colors of lesion and normal (CK) fruits were quantified. (**D**) Dihydrokaempferol, luteolin, rutin, (-)-catechin, naringenin chalcone, and quercetin contents of two colors of lesion and normal (CK) fruits were quantified. FW, fresh weight. Asterisk indicate significant differences: * *p* < 0.05, ** *p* < 0.01, *** *p* < 0.001.

To confirm the metabolite changes in tomato fruits during CYRSV infection, a carotenoid and flavonoid metabolomics assay was carried out using UPLC–MS/MS on different-colored lesions on tomatoes. As shown in the carotenoid metabolite enrichment analysis, three comparisons of differentially abundant metabolites, CYRSV (red-yellow) vs. CK (red), CYRSV (yellow) vs. CK (red), and CYRSV (yellow) vs. CYRSV (red-yellow), were performed, and the metabolites were coenriched in five pathways: carotenoid biosynthesis (ko00906), metabolic pathways (ko01100), the biosynthesis of secondary metabolites (ko01110), the biosynthesis of cofactors (ko01240), and the biosynthesis of various plant secondary metabolites (ko00999). Furthermore, the three comparisons with the greatest number of metabolites were enriched in carotenoid biosynthesis (ko00906), metabolic pathways (ko01100), and the biosynthesis of secondarymetabolites (ko01110) (Figure 4A).

The differentially abundant flavonoid metabolites in the three comparison groups, CYRSV (red-yellow) vs. CK (red), CYRSV (yellow) vs. CK (red), and CYRSV (yellow) vs. CYRSV (red-yellow), were enriched in five pathways: flavonoid biosynthesis (ko00941), flavone and flavanol biosynthesis (ko00944), metabolic pathways (ko01100), the biosynthesis of secondary metabolites (ko01110), and isoflavonoid biosynthesis (ko00943), with the highest number of metabolites enriched in the flavonoid biosynthesis pathway and the biosynthesis of secondary metabolites (Figure 4B). Metabolome analysis suggested that the biosynthesis of secondary metabolites (ko01110), carotenoid biosynthesis (ko00906), and flavonoid biosynthesis (ko00941) were strongly associated with the colored lesions induced by CYRSV.

To gain insights into the biochemical basis behind colored lesions, the levels and composition of carotenoids and flavonoids were determined in the fruits in the three different groups. The results of HPLC analysis revealed that the predominant carotenoids in the three groups of tomatoes were lycopene, α-carotene, β-carotene, and lutein. The levels of lycopene, α-carotene and β-carotene decreased during CYRSV infection, and the levels of lycopene and α-carotene decreased as the red color of the tomato lightened, whereas the lutein levels increased slightly (Figure 4C). In CYRSV-infected tomato fruits, the levels of dihydrokaempferol, luteolin, rutin, (-)-catechin, naringenin chalcone, and quercetin were greater in the CYRSV (yellow) group than in the CK (red) group (Figure 4D). These results suggest that the color lightening observed in the lesion was due to the absence of carotenoids (lycopene and α-carotene) and the accumulation of yellow-colored flavonoid metabolites induced by CYRSV in the fruit.

### 3.5. Transcriptome Analysis

**Figure 5:**
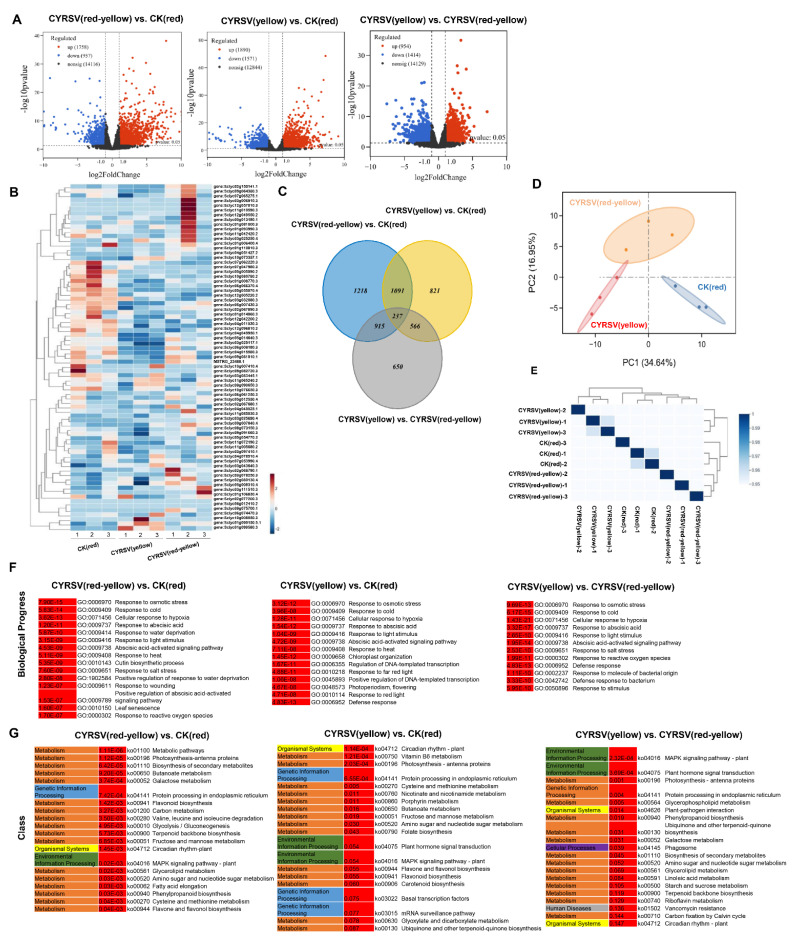
Transcriptome analysis. (**A**) Number of differentially expressed genes (DEGs) in CYRSV (red-yellow) vs. CK (red), CYRSV (yellow) vs. CK (red), and CYRSV (yellow) vs. CYRSV (red-yellow). (**B**) Clustering heatmap of carotenoid metabolites in the three comparisons. (**C**) Venn analysis results of different comparisons. (**D**) PCA distribution. (**E**) Sample correlation heatmap. (**F**) Biological process terms among the top 20 enriched GO terms for all the DEGs. (**G**) The top 20 enriched KEGG pathways for all the DEGs. Red indicates the *p* value, and the other colors indicate different classes of KEGG pathway.

To investigate the molecular mechanisms involved in metabolite regulation in tomato fruits infected with CYRSV, which results in colored lesions, we sequenced the transcriptomes of the three differently colored tomato fruits. The three comparisons were CYRSV (red-yellow) vs. CK (red), CYRSV (yellow) vs. CK (red), and CYRSV (yellow) vs. CYRSV (red-yellow), with 2715 (1758 upregulated and 957 downregulated), 3461 (1890 upregulated and 1571 downregulated), and 2368 (954 upregulated and 141 downregulated) DEGs, respectively (Figure 5A). In the thermogram, the genes in the CK (red), CYRSV (red-yellow), and CYRSV (yellow) groups presented similar accumulation trends (Figure 5B). Among all the DEGs, 1328 genes were differentially expressed in both the CYRSV (red-yellow) vs. CK (red) and CYRSV (yellow) vs. CK (red) comparisons, 1152 genes were differentially expressed in both the CYRSV (yellow) vs. CK (red) and CYRSV (yellow) vs. CYRSV (red-yellow) comparisons, 803 genes were differentially expressed in both the CYRSV (red-yellow) vs. CK (red) and CYRSV (yellow) vs. CYRSV (red-yellow) comparisons, and 237 genes were differentially expressed in all three comparisons (Figure 5C). As shown in Figure 5D, the principal component analysis (PCA) score plot clearly separated the CK (red), CYRSV (red-yellow), and CYRSV (yellow) samples. The heatmap revealed a relatively high correlation between biological replicates of the same group of samples, indicating good repeatability within the selected sample groups (Figure 5E). The results revealed that transcriptome changes were induced in tomato fruits with different-colored lesions under CYRSV infection.

The GO enrichment results revealed that numerous BP terms within the significant top 20 GO enrichment pathways were enriched in CYRSV (red-yellow) vs. CK (red) (15), CYRSV (yellow) vs. CK (red) (14), and CYRSV (yellow) vs. CYRSV (red-yellow) (12); these significant BP terms are associated with abiotic stress. However, the BP terms enriched in CYRSV (yellow) vs. CYRSV (red-yellow) are also associated with biotic stress, such as response to molecules of bacterial origin (GO:0002237) and defense response to bacteria (GO:0042742) (Figure 5F).

For KEGG enrichment analysis, we found that numerous pathways related to the “Metabolism” class among the top 20 significantly enriched KEGG pathways were enriched in CYRSV (red-yellow) vs. CK (red) (17), CYRSV (yellow) vs. CK (red) (14), and CYRSV (yellow) vs. CYRSV (red-yellow) (14). Among these, biosynthesis of secondary metabolites (ko01110), flavonoid biosynthesis (ko00941), phenylpropanoid biosynthesis (ko00940), and flavone and flavanol biosynthesis (ko00944) were significantly enriched in CYRSV (red-yellow) vs. CK (red). Flavonoid biosynthesis (ko00941), flavone and flavanol biosynthesis (ko00944), and carotenoid biosynthesis (ko00906) were significantly enriched in the CYRSV (yellow) vs. CK (red) comparison. The biosynthesis of secondary metabolites (ko01110) and of phenylpropanoids (ko00940) was significantly enriched in CYRSV (yellow) vs. CYRSV (red-yellow) (Figure 5G).

As shown in Table 1, the significant DEGs (*GGPS*, *NCED*, *CHS*, *CHI*, *FLS*, and *F3H*) enriched in the carotenoid biosynthesis and flavonoid biosynthesis pathways were significantly upregulated in CYRSV (red-yellow) vs. CK (red); the DEGs (*GGPS*, *PSY1*, *ZDS*, *LCYB*, *NCED*, *CHI*, *FLS*, and *F3H*) enriched in the carotenoid biosynthesis and flavonoid biosynthesis pathways were significantly upregulated in CYRSV (yellow) vs. CK (red); and the DEGs (*FLS* and *F3H*) enriched in the flavonoid biosynthesis pathway were significantly downregulated in CYRSV (yellow) vs. CYRSV (red-yellow). However, other genes related to the carotenoid biosynthesis and flavonoid biosynthesis pathways were not significantly enriched. Hence, both carotenoid biosynthesis and flavonoid biosynthesis are important pathways, which prompted us to elucidate the mechanism underlying the induction of tomato fruit lesions due to CYRSV.

### 3.6. Expression Analysis of Carotenoid and Flavonoid Metabolic Pathway Genes in Tomato Fruits with and Without Color Lesions

**Figure 6:**
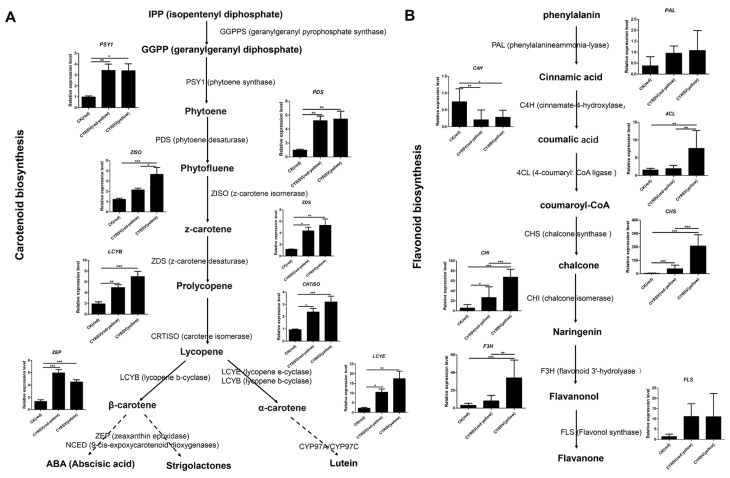
Schematic and gene expression profiling of the carotenoid and flavonoid biosynthetic pathways in tomato fruits infected with CYRSV. (**A**) Relative expression levels of key genes involved in the carotenoid metabolism pathway in tomato fruits with different-colored lesions. (**B**) Relative expression levels of key genes involved in the flavonoid metabolic pathway in tomato fruits with different-colored lesions. Asterisk indicate significant differences: * *p* < 0.05, ** *p* < 0.01, *** *p* < 0.001.

To elucidate the molecular basis behind the appearance of colored lesions following CYRSV infection, the same samples used for biochemical analysis were analyzed via RT–qPCR to determine the expression of several structural genes involved in carotenoid and flavonoid biosynthesis. A schematic of carotenoid and flavonoid synthesis is shown in Figure 6. In CYRSV-infected tomatoes, the expression of most of the key regulators, *PSY1*, *PDS*, *ZISO*, *ZDS*, *CRTISO*, *LCYB*, *LCYE*, and *ZEP*, was greater in both CYRSV (red-yellow) and CYRSV (yellow) than in the CK (red). Among them, the expression of the *ZISO*, *ZDS*, *CRTISO*, *LCYB*, and *LCYE* genes was greater in the CYRSV (yellow) group than in the CYRSV (red-yellow) group, with or without significant differences. The expression of the five genes increased in a CYRSV accumulation-dependent manner (Figure 6A). In CYRSV-infected tomatoes, the mRNA levels of the major flavonoid pathway genes *PAL*, *4CL*, *CHS*, *CHI*, *F3H*, and *FLS* expressed in tomato fruits were high, and the mRNA levels of *4CL*, *CHS*, *CHI*, and *F3H* were significantly higher in the CYRSV (yellow) group than in the CYRSV (red-yellow) group. The expression of these four genes also increased in a CYRSV accumulation-dependent manner. In contrast, *C4H* levels decreased strongly in CYRSV-infected tomatoes (Figure 6B). The above results were essentially consistent with the RNA-Seq results, and the discrepancies at different developmental stages between the RT–qPCR and RNA–Seq results might have been caused by a sensitivity bias between the two methods and sampling differences in the same tomato sample.

### 3.7. CYRSV Accumulation Is Associated with the Expression of Genes Encoding the Key Enzyme in Carotenoid and Flavonoid Pathways

**Figure 7:**
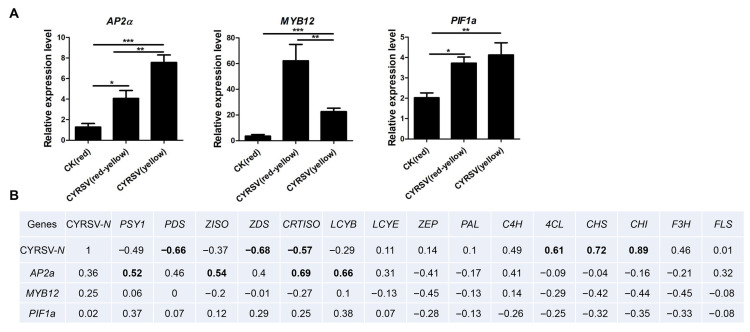
Expression of the pigment regulating transcription factors *AP2a*, *MYB12*, and *PIF1a* in tomato fruits infected with CYRSV. (**A**) Relative expression of *AP2a*, *MYB12*, and *PIF1a* in harvested tomatoes with different-colored lesions determined via RT–qPCR. (**B**) Correlation analysis of the carotenoid and flavonoid gene expression profiles with the expression levels of the *AP2a*, *MYB12*, and *PIF1a* genes. Black bold font represents significant correlation. Asterisk indicate significant differences: * *p* < 0.05, ** *p* < 0.01, *** *p* < 0.001.

As shown in Table 2, the levels of *MYB12* and *PIF1* were greater in both CYRSV (red-yellow) and CYRSV (yellow) than in CK (red). However, compared with that in the CK (red) group, the expression of some members of the AP2/ERF family in the CYRSV (red-yellow) group increased, while that of some other members did not significantly differ (Table 2). The AP2/ERF family, the MYB family, and *PIF1a* are related to the regulation of carotenoid synthesis and flavonoid synthesis. On the basis of the results of the transcriptome analysis and the literature, the possible roles of transcription factors (*AP2a*, *MYB12*, and *PIF1a*) in regulating the production of carotenoids and flavonoids in CYRSV-infected tomatoes were analyzed via RT–qPCR. The transcription factors *AP2a*, *MYB12*, and *PIF1a* were highly significantly expressed in tomato fruits with colored lesions (Figure 7A). The observed gene expression patterns were subsequently compared with those of the carotenoid and flavonoid genes. Correlation analysis revealed that the expression of CYRSV-*N* was negatively correlated with the expression of carotenoid genes (*PDS*, *ZDS*, and *CRTISO*) and positively correlated with the expression of flavonoid genes (*4CL*, *CHS*, and *CHI*), suggesting that the carotenoid and flavonoid genes are potential colored-lesion regulators in CYRSV-infected tomato fruit. Moreover, the expression of *AP2a* was positively correlated with the expression of genes encoding the key enzyme of carotenoids (*PSY1*, *ZISO*, *CRTISO*, and *LCYB*), suggesting that *AP2a* is a potential regulator of carotenoid biosynthesis in tomato fruits (Figure 7B). Our findings suggest that CYRSV influences *AP2a* levels to activate genes encoding enzymes of the carotenoid pathway to regulate the production of carotenoids in tomato fruits, which leads to colored lesions in tomato fruits.

### 3.8. Effects of CYRSV Infection on Tomato Fruit Color, Chromoplast Ultrastructure, and Carotenoid and Flavonoid Pathways

**Figure 8:**
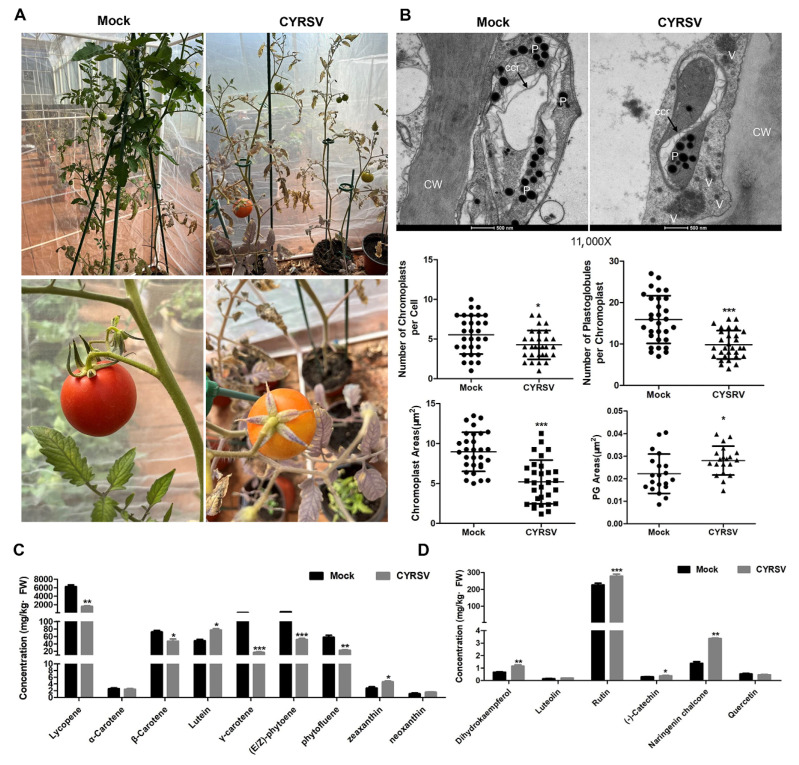
Effects of CYRSV infection on the color, chromoplasts, and carotenoid and flavonoid pathways in tomato fruits. (**A**) Tomato symptoms after CYRSV infection. (**B**) Chromoplast micrographs for the CYRSV and mock groups. Numbers and areas of chromoplasts and plastoglobules in the CYRSV and mock groups. P, plastoglobule; ccr, carotenoid crystalloid; CW, cell wall; V, virus; scale bar = 500 nm. Carotenoid (**C**) and flavonoid (**D**) metabolite contents in tomato fruits from the CYRSV and mock groups. Asterisk indicate significant differences: * *p* < 0.05, ** *p* < 0.01, *** *p* < 0.001.

To further investigate the relationship between CYRSV and colored lesions, an experiment involving CYRSV inoculation in tomato plants was performed. As shown in Figure 8A, the tomato plants in the mock group thrived, but some symptoms were detected, such as dwarfing, purple veins in leaves, and yellowing of fruits in the CYRSV group. CYRSV-infected fruits presented a lower number of chromoplasts and plastoglobules and a decreased area of chromoplasts but an increased area of plastoglobules (Figure 8B). The contents of carotenoids (lycopene, α-carotene, β-carotene, and lutein) were subsequently measured via UPLC analysis, and the levels of lycopene, α-carotene, and β-carotene markedly decreased during CYRSV infection, whereas that of lutein slightly increased (Figure 8C). Moreover, the contents of flavonoids (dihydrokaempferol, rutin, (-)-catechin, and naringenin chalcone) significantly increased during CYRSV infection, whereas the difference between the levels of luteolin and quercetin was not significant (Figure 8D). Most of these results are consistent with those of our previous experiments.

## 4. Discussion

Viral infection in a plant can lead to changes in fruit color, and the color of the fruit determines its commercial value. The color of a fruit is considered the most important external feature for fruit selection, harvest, and sale [24]. A lack of color uniformity can represent a significant problem in the production and marketing of important commercial crops. In recent years, CYRSV has spread and proliferated among tomatoes in Yuanmou, Yunnan Province [19]. CYRSV induces colored lesions in tomato fruits, resulting in significant economic losses in agricultural production. The mechanism by which viruses cause colored lesions in fruits remains unclear. Thus, studying the related mechanisms of tomato fruit color lesions caused by viral infection is highly important in the prevention and control of viral tomato diseases.

In this study, we collected CYRSV-infected ‘Liangsi’ tomato fruits with red-yellow and yellow lesions as experimental materials for studying ultrastructural pathological changes in chromoplasts. Comparative analysis of carotenoid and flavonoid metabolism and transcriptomes was performed to determine the material composition associated with fruit color. We first measured virus accumulation in tomato fruits with different-colored lesions using an absolute quantitative qPCR assay. The qPCR results suggested that CYRSV infection could lead to different-colored lesions in fruits at the ripening stage, which were associated with the accumulation of CYRSV viral particles; a greater degree of virus accumulation resulted in a lighter fruit color. We subsequently analyzed the ultrastructural characteristics of the chromoplasts of tomato fruits with different-colored lesions. We found that both the number of plastoglobules and the chromoplast areas markedly decreased, whereas the plastoglobule areas markedly increased, in tomato fruits with different-colored lesions. Chromoplasts accumulate massive amounts of carotenoids and possess a superior storage capacity, allowing them to deposit carotenoids in carotenoid–lipoprotein-sequestering structures and/or plastoglobules [25]. Plastoglobules are potentially involved in chromoplast biogenesis [25]. In addition to their well-established function in carotenoid sequestration and storage [26], plastoglobules are known to have the capacity to synthesize carotenoids [27]. These structures increase the sink strength of chromoplasts, leading to a massive accumulation of carotenoids in many flowers, fruits, and vegetables. Our results are consistent with those of these previous studies, indicating that CYRSV causes damage to the structure of chromoplasts, which in turn leads to changes in the accumulation of carotenoids and ultimately results in fruit color disorders. The color of tomato fruits is related to the contents of carotenoid and flavonoid metabolites.

Carotenoids are synthesized in plants through different metabolic processes with different biological functions [28,29]. Carotenoids have diverse functions in terms of antioxidant activity, light trapping, photoprotection, the prevention of degenerative diseases such as atherosclerosis and cancer, and slowing down aging [30,31]. Flavonoids are secondary metabolites that are widely found in plants and are commonly present in foods that people consume regularly, such as fruits, vegetables, and cereals [32,33,34]. The color transformation of fruits such as tomatoes and citrus fruits is associated with flavonoids [35,36,37]. The results of our carotenoid and flavonoid metabolite analysis in tomato fruits with different-colored lesions revealed that red-yellow and yellow fruits had lower lycopene and α-carotene contents and that yellow fruits presented the greatest accumulation of yellow-colored flavonoid metabolites, such as naringenin chalcone, among fruits with different-colored lesions. Our data indicate that the types of metabolites that are significantly enriched differ for different-colored lesions in tomato fruits, with carotenoids, flavonoids, flavanols, and flavones being the major metabolites involved in tomato fruit discoloration. These results indicated that CYRSV suppresses the accumulation of carotenoid metabolites (lycopene and a-carotene) and promotes the accumulation of flavonoid metabolites, which leads to changes in color in tomato fruits. According to the results of the RNA-seq and qPCR analyses, “carotenoid biosynthesis” and “flavonoid biosynthesis” are important pathways that regulate the mechanism behind the induction by CYRSV of colored lesions in tomato fruits. The qPCR results were essentially consistent with the RNA–Seq results, and the discrepancies at different developmental stages between the RT–qPCR and RNA–Seq results may have been caused by a sensitivity bias between the two methods and sampling differences within the same tomato sample.

Carotenoids are regulated by a class of transcription factors belonging to the AP2/ERF and MYB families in plants [38,39]. Flavonoid pathways are regulated by a class of transcription factors belonging to the R2R3MYB family in plants [40,41]. Phytochrome-interacting factors (PIFs), which belong to the bHLH superfamily of transcription factors and are localized in the nucleus, can influence carotenoid accumulation [42,43,44]. We selected candidate transcription factor genes (AP2a, MYB12, and PIF1a) that are involved in regulating the carotenoid and flavonoid pathways [45,46,47,48,49,50]. A total of three candidate transcription factors (AP2a, MYB12, and PIF1a) were selected, and their expression in fruits with different-colored lesion-related genotypes was determined via RT–qPCR. The gene levels of the main carotenoid and flavonoid pathways and these transcription factors were affected in the red-yellow and yellow fruit samples, suggesting that CYRSV affects both the carotenoid pathway and the flavonoid pathway, as well as these transcription factors. AP2a expression was markedly induced in tomato fruits during CYRSV infection, but the levels of carotenoid metabolites (lycopene and a-carotene) significantly decreased in tomato fruits during CYRSV infection. The expression of one transcription factor gene, AP2a, was positively correlated with the expression of all biosynthetic carotenoid genes in red-yellow and yellow-colored tomato fruits, suggesting that this gene may be a direct regulator of the carotenoid pathway in tomato fruits. Recently, the overexpression of the *AtMYB12* gene in tomato fruit was shown to strongly induce flavonoid and phenylpropanoid gene expression and strongly increase the levels of flavonols and hydroxycinnamic acids, particularly chlorogenic acid, in both the fruit peel and flesh [51]. In this study, after CYSRV infection, MYB12 expression was induced in both red-yellow and yellow tomato fruits compared with the CK group. However, MYB12 expression was markedly induced in red-yellow tomato fruits, while the levels of flavonoid metabolites (dihydrokaempferol, luteolin, rutin, catechin, naringenin chalcone, and quercetin) were significantly increased in yellow tomato fruits. Moreover, MYB12 expression was negatively correlated with the expression of biosynthetic flavonoid genes, except for the *C4H* gene, in red-yellow and yellow tomato fruits.

Greenhouse isolation and inoculation experiments were performed to verify that the colored lesions caused by CYRSV in tomato fruits were related to the chromoplasts and the carotenoid and flavonoid pathways. Similarly, CYRSV infection led to yellow fruit in tomato plants and decreased the number of chromoplasts/plastoglobules, the chromoplast area, and carotenoid metabolite levels while increasing the plastoglobule area and flavonoid metabolite levels.

## 5. Conclusions

In conclusion, the colored lesions on the CYRSV-infected tomato fruits analyzed in this study could be attributed to changes in the carotenoid and flavonoid metabolism pathways; “carotenoid biosynthesis” and “flavonoid biosynthesis” are important pathways that regulate the colored lesions on tomato fruits induced by CYRSV. In CYRSV-infected tomatoes, we detected reduced levels of lycopene and α-carotene, key metabolites in the carotenoid biosynthetic pathway, but increased levels of rutin, catechin, and naringenin chalcone, key metabolites in the flavonoid biosynthetic pathway. These results suggest that the red-yellow and yellow color of CYRSV-infected tomato fruits is due to a decreased number of plastoglobules, decreased chromoplast area, the absence of carotenoid metabolites (lycopene and α-carotene), the presence of yellow-colored flavonoid metabolites (rutin, catechin, and naringenin chalcone), and increased expression of AP2a/MYB12, a transcription factor gene that regulates the carotenoid and flavonoid pathways.

Colored lesions induced by CYRSV in tomato fruits constitute a complex multiomics and multimaterial interaction process, and further investigations are needed to fully understand the molecular mechanisms and principles involved in each of these steps. From an application perspective, our findings provide a theoretical basis for screening disease resistance genes in future disease resistance breeding programs. Furthermore, our results provide new insights into the mechanisms behind the interaction of genes and metabolites involved in the biosynthesis of flavonoids and carotenoids in tomato fruits that lead to colored lesions in CYRSV-infected tomatoes.

## Figures and Tables

**Table 1:** Expression of genes of interest in carotenoid and flavonoid biosynthesis pathways identified in all comparisons.

Gene ID	Gene Description	Gene Name	CYRSV (Red-Yellow) vs. CK (Red)	CYRSV (Yellow) vs. CK (Red)	CYRSV (Yellow) vs. CYRSV (Red-Yellow)
Solyc02g085710.4	geranylgeranyl pyrophosphate synthase	*GGPS*	up	up	/
Solyc03g031860.3	phytoene synthase 1	*PSY1*	ns	up	/
Solyc03g123760.3	15-cis-phytoene desaturase	*PDS*	ns	ns	/
Solyc12g098710.2	15-cis-zeta-carotene isomerase	*ZISO*	ns	ns	/
Solyc01g097810.3	zeta-carotene desaturase	*ZDS*	ns	up	/
Solyc04g040190.1	lycopene beta cyclase	*LCYB*	ns	up	/
Solyc02g090890.4	zeaxanthin epoxidase	*ZEP*	ns	ns	/
Solyc07g056570.1	9-cis-epoxycarotenoid dioxygenase	*NCED*	up	up	/
Solyc08g076300.3	4-coumarate-CoA ligase-like 6	*4CL*	ns	ns	/
Solyc09g091510.3	chalcone synthase 1	*CHS*	up	ns	/
Solyc05g052240.3	probable chalcone-flavonone isomerase 3	*CHI*	up	up	/
Solyc11g013110.2	flavonol synthase/flavanone 3-hydroxylase	*FLS/F3H*	up	up	down

ns, no significant difference (*p* > 0.05).

**Table 2:** Expression of the genes of interest among the transcription factors identified in all the comparisons.

Gene ID	Gene Description	Gene Name	CYRSV (Red-Yellow) vs. CK (Red)	CYRSV (Yellow) vs. CK (Red)	CYRSV (Yellow) vs. CYRSV (Red-Yellow)
Solyc02g093130.3	AP2/ethylene-responsive transcription factor	*RAP2-10*	up	up	/
Solyc07g054220.1	AP2/ethylene-responsive transcription factor	*ERF054*	ns	up	up
Solyc01g079620.4	Myb12 transcription factor	*MYB12*	up	up	ns
Solyc09g063010.4	transcription factor PIF1	*PIF1*	up	up	up

ns: no significant difference (*p* > 0.05).

## Data Availability

Data are contained within the article.
